# A Road Map for the Global Elimination of Congenital Syphilis

**DOI:** 10.1155/2010/312798

**Published:** 2010-07-14

**Authors:** Mary L. Kamb, Lori M. Newman, Patricia L. Riley, Jennifer Mark, Sarah J. Hawkes, Tasneem Malik, Nathalie Broutet

**Affiliations:** ^1^Division of STD Prevention (DSTDP), International Activities Unit, U.S. Centers for Disease Control and Prevention (CDC), 1600 Clifton Rd, NE, MS E-02, Atlanta, GA 30333, USA; ^2^Department of Reproductive Health and Research, World Health Organization (WHO), Geneva, Switzerland; ^3^Division of Global HIV/AIDS, CDC, Atlanta, GA, USA; ^4^University College London, London, UK

## Abstract

Congenital syphilis is the oldest recognized congenital infection, and continues to account for extensive global perinatal morbidity and mortality today. Serious adverse pregnancy outcomes caused by maternal syphilis infection are prevented with screening early in pregnancy and prompt treatment of women testing positive. Intramuscular penicillin, an inexpensive antibiotic on the essential medicine list of nations all over the world, effectively cures infection and prevents congenital syphilis. In fact, at a cost of $11–15 per disability adjusted life year (DALY) averted, maternal syphilis screening and treatment is among the most cost-effective public health interventions in existence. Yet implementation of this basic public health intervention is sporadic in countries with highest congenital syphilis burden. We discuss the global burden of this devastating disease, current progress and ongoing challenges for its elimination in countries with highest prevalence, and next steps in ensuring a world free of preventable perinatal deaths caused by syphilis.

## 1. Introduction

Mother-to-child transmission of syphilis, that is, congenital syphilis, has been documented since the 15th century [1], yet continues today to cause substantial perinatal morbidity. If left untreated, maternal syphilis infection will, in up to 80% of pregnancies, lead to severely adverse pregnancy outcomes including stillbirth, premature birth, neonatal death, or congenital infection in the newborn [2]. In light of continuing perinatal mortality caused by syphilis and the high cost-effectiveness of antenatal screening and treatment as an intervention package [3], in 2007 the World Health Organization (WHO) launched a global initiative for the elimination of congenital syphilis. In subsequent analyses, WHO has estimated that, were ten countries with high antenatal syphilis burden allowed to focus efforts in strengthening existing maternal and child health (MCH) systems infrastructure to ensure universal maternal syphilis screening coupled with prompt treatment, an investment of only $3 to 4 million dollars per year over five years could substantially reduce this global perinatal scourge. In addition to these resources, elimination of congenital syphilis requires a combined commitment of governments and other partners in order to mount an effective and sustained response. The global elimination of congenital syphilis can greatly support current global efforts, including those outlined in the Millennium Development Goals of reducing child mortality, improving maternal health, and combating HIV, malaria and other infectious diseases. 

## 2. Global Burden of Congenital Syphilis

Globally just over 2 million pregnant women test positive for syphilis each year, comprising 1.5 percent of all pregnancies worldwide [9, 10]. *Treponema pallidum*, the bacteria causing syphilis, is able to traverse the placenta early in pregnancy and lead to fetal exposure; however, fetal compromise is generally not manifested until later in the second or third trimesters with maturation of the fetal immune system [2]. Cohort studies have been consistent in finding that substantial proportions (40–81 percent) of syphilis-exposed fetuses are severely affected, with stillbirth neonatal death being the most significant consequences (Table 1) [4–8]. A smaller but substantial proportion of untreated maternal infections results in congenital infection in newborn infants, often manifested by premature birth, low birth weight, and failure to thrive. Among surviving infants, visceral involvement is common, as are fever, rashes, blindness, and a variety of typical skeletal and dental abnormalities [1].

 The World Health Organization (WHO) estimates that globally the majority of maternal syphilis infections are untreated and of sufficiently high-titer (RPR ≥ 1 : 8) to cause significant fetal exposure to *T. pallidum. *This situation results in an estimated 692,100 to 1.53 million adverse pregnancy outcomes each year caused by syphilis (Table 2) [8, 11–13]. Approximately 650,000 of these pregnancy complications result in perinatal deaths (i.e., deaths occurring from 22 weeks gestation through the first 7 days of life). Thus, untreated maternal syphilis is believed to have at least similar mortality, if not higher, than other important infections during pregnancy such as HIV (estimated to cause 250,000–290,000 perinatal deaths globally) [14] neonatal tetanus (200,000 perinatal deaths), or malaria in pregnancy (200,000 perinatal deaths) [13]. Globally, untreated maternal syphilis infection accounts for up to one quarter of all stillbirths and 11% of neonatal deaths, with most of these perinatal deaths occurring in developing settings with moderate or high antenatal syphilis prevalence and weak health systems [15]. 

## 3. Situation: Health Services Delivery Now

Adverse pregnancy outcomes caused by syphilis can be almost entirely averted through early identification of maternal infections through antenatal screening programmes, and prompt treatment of women with positive tests with a single dose of long acting penicillin prior to 24 weeks gestation [16]. Maternal syphilis screening and treatment are recognized as part of essential antenatal services [17], and almost all nations already have existing policies recommending universal syphilis screening during pregnancy [18]. However, implementation of the policy is weak in many settings, particularly in countries with highest disease burden. 

 High-burden countries have reported numerous barriers that limit effective screening and treatment as part of basic antenatal health services [19]. Access to early antenatal clinical (ANC) services, especially prior to 24 weeks gestation, is still limited in many parts of the world, and particularly in sub-Saharan African nations where a third of women either receive care later in pregnancy or do not receive care at all [17]. Health providers, particularly those in lower-level health facilities, may be unaware of the burden of congenital syphilis or the need to identify and treat early in pregnancy, and thus may not prioritize syphilis screening for the first antenatal visit. Syphilis diagnosis can be complicated and usually requires serologic screening with nontreponemal (e.g., RPR) or treponemal (e.g., TPHA) tests, and ideally both. While RPR tests, measuring active infection, are relatively simple and inexpensive, they require basic laboratory capacity, trained technical staff, and ongoing quality control systems. Unfortunately many lower-level facilities providing antenatal services have *no* laboratory access, thus testing is often simply not done at all. In antenatal facilities with access to laboratories able to conduct RPR tests (typically district level or higher facilities), women must often go to a separate site for testing, incurring additional transportation costs and waiting time above the basic antenatal visit. Additionally, women are often asked to pay for the tests themselves, a substantial barrier for many women [17, 20–23]. 

 Even in places that could feasibly provide testing routinely, further systems level issues such as stockouts of critical commodities and inadequate numbers or distribution of clinical or laboratory providers can limit testing. Also, for women with positive tests, treatment can be delayed when results are not provided in a timely fashion, or when treatment is difficult to access. Women with positive screening tests may not learn about positive results until a later clinic visit, and are often asked to travel to another site for treatment—for which they often, again, have to pay themselves. Furthermore, while most programmes collect data on maternal screening, few collect data on provision or timing of treatment, and thus programmes may be unaware of their deficiencies in providing prompt and appropriate treatment [22, 23]. Another increasingly reported barrier that limits programme coverage and effectiveness is the provision of disease-specific antenatal care. In some settings women are required to attend different clinics for basic ANC services, HIV testing and prevention of mother to child transmission services (PMTCT), and malaria prevention and treatment, for example [23]. 

## 4. WHO Initiative for the Global Elimination of Congenital Syphilis

### 4.1. Rationale and Strategy

Taking into consideration the current challenges to congenital syphilis prevention, WHO has outlined a strategic plan of action for the global elimination of congenital syphilis [20] as a public health problem. Given difficulties in diagnosing and monitoring syphilis-related complications, the specific goal of this elimination effort is to prevent transmission of syphilis from mother to child. This can be achieved by strengthening antenatal care programmes to ensure that all women receive early antenatal care which includes universal syphilis screening, prompt and appropriate treatment, and counselling on how to prevent infection. Additionally, recommendations that partners of infected women are treated (reducing reinfection) and that all neonates born to infected mothers are treated will help reduce congenital syphilis in live borne infants. 

 WHO has also outlined a series of guiding principles upon which the global congenital syphilis elimination strategy was developed: *country-driven* to adapt to local needs; *integrated* to ensure that the effort strengthens existing STI, HIV, prenatal, and maternal and newborn health services; *rights-based* to ensure that all individuals have the knowledge to participate in decision-making about their health and access to high-quality care, and *collaborative* so that government bodies, donors, and communities work together to optimize use of scarce resources.

**Figure 1:**
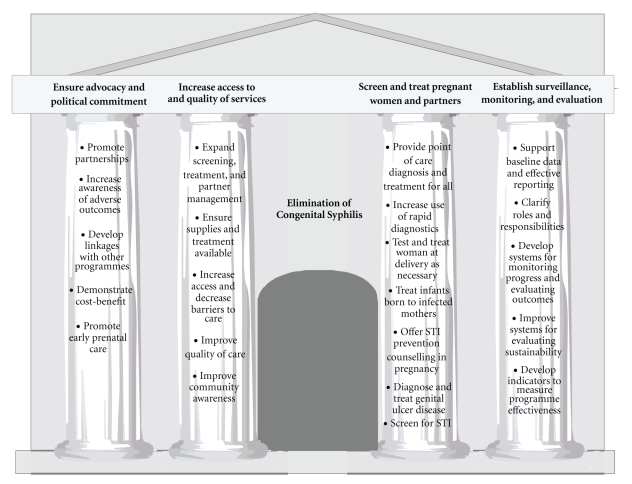
The four pillar strategy for elimination Congenital Syphilis.

At a country level, WHO outlined a strategy consisting of four pillars with corresponding specific objectives of actions to be undertaken (Figure 1). The pillars are the following:

1. ensure advocacy and sustained political commitment for a successful health initiative,
2. increase access to, and quality of, maternal and newborn health services,
3. screen and treat pregnant women and partners, and
4. establish surveillance, monitoring, and evaluation systems.

The global initiative emphasizes that congenital syphilis elimination can contribute directly to three of the Millennium Development Goals (MDGs) by reducing child mortality (MDG4) through reductions in perinatal deaths and low-birth-weight infants; improving maternal health (MDG5) through reductions in late fetal losses and stillbirths and through a decreased burden of syphilis in pregnant women, and combating HIV/AIDS, malaria and other diseases (MDG6) through combined, systematic screening for HIV and syphilis in pregnancy with an emphasis on strengthening antenatal and postpartum health systems.

### 4.2. Measuring National and Local Programme Progress and Impact

Although some countries routinely monitor reports of congenital syphilis as a routine part of public health reporting, in general, case reporting of congenital syphilis is problematic as its definitive diagnosis is not easy. To address this, a WHO-led working group has identified a set of outcome and process indicators that together are feasible measures of programme progress and impact on congenital syphilis elimination. Monitoring the proportion of stillbirths attributable to syphilis was identified by the working group as the most promising outcome indicator, and it was recommended that a target for nations should be that “the proportion of stillbirths attributable to syphilis in the mother be less than 2 percent.” 

 The stillbirth target of 2 percent was chosen because stillbirth is both the most common and most severe outcome caused by untreated maternal syphilis. Among women with active syphilis, 17 to 40 percent of pregnancies result in stillbirth, and the risk of stillbirth has been reported as ten to 18 times the background rate of stillbirth (approximately 2 percent) [4–8]. In settings with moderate to high maternal syphilis prevalence, congenital syphilis has been reported to account for more than 20 percent of all stillbirths (i.e., attributable fraction) [7, 8]. The 2 percent target was chosen as both an aspirational benchmark and one that, historically, was able to be achieved in settings adopting universal syphilis screening in pregnant women and prompt treatment of those testing positive [5, 6]. 

 Three critical process indicators were also identified to monitor programme progress, all involving collection of local data. These are (1) the proportion of women tested for syphilis at their first antenatal care visit, (2) the proportion of pregnant women with a positive test for syphilis, and (3) the proportion of positive women treated for syphilis, ideally by 24 weeks. These few process indicators, along with currently collected indicators on estimated number of pregnancies and coverage of antenatal screening, potentially allow countries to calculate a summary process indicator that estimates overall programme effectiveness, that is “the estimated proportion of all syphilis-positive pregnant women treated by 24 weeks of gestational age.” This indicator is important for countries to ascertain since treatment sufficiently early in pregnancy (prior to 24 to 28 weeks) is necessary to avert the adverse effects of syphilis in pregnancy in most situations [8]. 

## 5. Call to Action: An Investment Case for Eliminating Congenital Syphilis

In order to raise funds for the goal of eliminating congenital syphilis, WHO and its partners have developed an Investment Case for resource mobilization and to raise awareness of the issue and the proposed solutions. Cost calculations within the Investment Case have highlighted the significant DALY burden associated with untreated syphilis in pregnant women and have demonstrated how this DALY burden disproportionately affects people in low- and middle-income countries. The Investment Case shows how the elimination of congenital syphilis is a relatively “easy win” for programmes in affected countries as the intervention is relatively simple, highly cost-effective, technically feasible, and already politically acceptable (as demonstrated by consistent policies already in existence). Moreover, the Investment Case stresses that elimination of congenital syphilis is timely for countries that are aiming to reach their Millennium Development Goal commitments by 2015. 

## 6. Next Steps

With sufficient resources, several additional areas can be better addressed to ensure that public health efforts aimed at the global elimination of congenital syphilis are effective, sustainable, and support overall MCH systems strengthening. 

### 6.1. Addressing Barriers and Ensuring Sustainability of Programmes

As noted, a number of barriers currently exist that limit maternal syphilis screening and treatment efforts in developing world settings. Despite this situation, evidence based on current programmes supports that many of these barriers can be effectively countered if resources and efforts focus on antenatal health systems strengthening [22, 23]. An important component of the global initiative to eliminate congenital syphilis is that it promotes maternal syphilis screening and treatment as part of basic antenatal health services, thus is framed as a means of strengthening overall MCH systems rather than only battling congenital syphilis. In its promotion of early antenatal care with the recommended basic package of health services, the initiative intervenes against a range of preventable causes of perinatal morbidity and mortality. Similarly, in promoting integration of ANC service delivery through strategies such as incorporating integrated professional training and curricula for health care providers, coordinating distribution systems for critical commodities around ANC services, or integrating data systems monitoring, the initiative supports building capacity for improved antenatal outcomes overall. 

 Health service research studies have identified that another means of ensuring sustainability of programmes is through decentralization of laboratory services, such as provision of same day testing and treatment through rapid point-of-care tests [17, 20–23]. Several such tests that are heat stable, easy to use, and low cost ($0.19–0.99 per test) have been found to be sensitive and specific in very basic clinic settings [21, 22]. Use of such point-of-care tests ensures women with positive results are treated as early as possible, minimizing loss to followup and maximizing the potential to avert pregnancy complications. Currently available point-of-care syphilis tests are all treponemal tests, identifying any prior infection (even previously treated cases) as opposed to active infection. This can result in overtreatment of women whose prior infections were already treated. Nonetheless, studies conducted in antenatal settings lacking sufficient laboratory capacity for RPR testing have found introduction of point-of-care tests greatly enhanced screening rates [22–24], was acceptable and had negligible risks for women [23, 24], and was highly cost-effective even taking into account some overtreatment [24]. 

 Another commonly reported barrier has been a requirement that screening and treatment costs are borne by the patient. Provision of free-of-charge or low-cost testing and treatment through government programmes has proven important in some settings to ensure the highest risk women are effectively screened and, if positive, treated [23]. This policy has been found to support health worker's ability to provide syphilis screening as a routine part of antenatal care, solidifying the habit of universal screening, also important in sustainability [23]. 

 An especially difficult barrier to surmount in many settings is ensuring adequate human resources. In particular, shortfalls in clinical faculty and the absence of training and student mentoring opportunities can complicate strategies for eliminating congenital syphilis. As a result, medical, nursing and midwifery trainees not only lack updated professional curricula and learning materials (e.g., on rapid, point-of-care treponemal tests and syphilis treatment algorithms) but also lack practical experience. Addressing this requires the inclusion of current curricula and protocols within pre-service education as well as taking opportunities to observe and provide holistic antenatal care in which syphilis prevention and treatment are incorporated into service delivery. Equally important is engaging national accreditation and credentialing bodies in efforts focused on the elimination of congenital syphilis. An additional opportunity for influencing provider practice is through partnering with global professional associations, such as, the International Federation of Gynecology and Obstetrics (FIGO), the International Confederation of Midwives (ICM) and the International Council of Nurses (ICN). Such organizations are vested in advancing global standards of care within their respective profession and can greatly influence syphilis prevention (and its appropriate adaptation) in high-burden countries. 

### 6.2. Health Systems Strengthening

Supporting the creation of strong, sustainable health systems that are responsive, efficient and equitable is now recognized as a key component of improving health for all [25–27]. The 2005 “Paris Declaration on Aid Effectiveness” emphasizes such health systems strengthening through local ownership (of the intervention), support of local health systems, harmonization of donor investment (thereby avoiding duplication), results measurement, and agreement of mutual accountability for attainment of development objectives [28]. Similar with other health initiatives, WHO has addressed this in some detail in the four pillar strategy identified for the global initiative for the elimination of congenital syphilis [20]. 

 However, an inherent challenge lies in striking an appropriate balance between achieving programme and disease-specific goals while at the same time ensuring long-term sustainability through well-functioning and adequately-resourced health systems. This challenge also provides an opportunity in that investments that strengthen health systems can be leveraged to ensure that, in nations with high perinatal morbidity, congenital syphilis interventions are part of the comprehensive MCH systems strengthening efforts. Similarly, partnering with other global health initiatives such as the Global Fund (To Fight AIDS, Tuberculosis and Malaria) [25] and the President's Emergency Plan for AIDS Relief (PEPFAR) [26] can help support congenital syphilis interventions that are provided to the same at-risk population. The shared goals of improved infant and maternal health encompass the areas of service delivery, healthy workforce development, information needs, medical products and technologies, financing, leadership, and governance [27]. 

## 7. Summary

Untreated maternal syphilis infection continues to account for large numbers of perinatal deaths worldwide, primarily in nations with moderate to high community prevalence of syphilis and weak health systems. These perinatal deaths are preventable with sufficiently early antenatal syphilis screening and prompt treatment of women testing positive. This highly cost-effective intervention has been demonstrated to be successfully achieved in several high-burden settings through strengthening current health systems. Now—in the context of Millennium Development Goals aimed at promoting infant health and averting preventable deaths—is a prime opportunity to address this old disease through promoting stronger antenatal health systems. 

 The 2007 WHO initiative for the global elimination of congenital syphilis provides a framework for this effort. The monitoring and evaluation plan developed with support from representatives of high-burden nations provides a means of ensuring progress and accountability through enhancing existing data systems. In addition, the Investment Case developed by WHO and partners offers a potential to identify the resources needed to achieve congenital syphilis elimination over the next five years in ten of the world's highest burden countries. Evaluations of current programmes have helped identify several of the existing barriers to current maternal syphilis screening and treatment efforts as well as potential solutions to address them. Key among these is a need to support overall health systems strengthening. Now remains the important step of bringing this effort to fruition through integration with other programmes, often disease-specific efforts, aimed at reducing maternal and infant morbidity and mortality. Effective partnerships, provider training, and empowerment can help support this critical process aimed at improving overall infant health.

## Figures and Tables

**Table 1:** Adverse pregnancy outcomes due to maternal syphilis.

Outcome	Harmon [4]	Ingraham [5]	Rabut [6]	McDermott [7]	Watson-Jones [8]
	(*N* = 1001)	(*N* = 302)	(*N* = 722)	(*N* = 436)	(*N* = 100)
Stillbirth	17%	22%	36%	46%	25%
Neonatal Death	23%	12%		35%	NA
Prematurity or low birth weight	NA	NA	NA	NA	25%
Infected Infant	21%	33%	NA	NA	NA
Any adverse outcome	61%	67%	36%	81%	49%

NA = Data not assessed.

**Table 2:** Estimated global burden of congenital syphilis cases.

Proportion of seropositive women with:	Watson-Jones [8, 11]	Schulz [12]	WHO [13]
Untreated syphilis	95%*	100%	100%
High serologic titer (≥1 : 8)	73%	—	—
Adverse pregnancy outcome due to syphilis**	49%	65%	75%
Global Annual No. of Congenital Syphilis Cases	692,100	1,323,900	1,527,600

Note that all assumptions based on WHO estimate of 2,036,753 pregnant women with syphilis [9].

*not included in the original Watson-Jones model; **includes late fetal loss, perinatal death, prematurity/low birth weight, neonatal infection.
